# Contribution of human and climate change impacts to changes in streamflow of Canada

**DOI:** 10.1038/srep17767

**Published:** 2015-12-04

**Authors:** Xuezhi Tan, Thian Yew Gan

**Affiliations:** 1 Department of Civil and Environmental Engineering, University of Alberta, Edmonton, Alberta, Canada T6G 2W2; 2 State Key Laboratory of Water Resources and Hydropower Engineering Science, Wuhan University, Wuhan, China 430072

## Abstract

Climate change exerts great influence on streamflow by changing precipitation, temperature, snowpack and potential evapotranspiration (PET), while human activities in a watershed can directly alter the runoff production and indirectly through affecting climatic variables. However, to separate contribution of anthropogenic and natural drivers to observed changes in streamflow is non-trivial. Here we estimated the direct influence of human activities and climate change effect to changes of the mean annual streamflow (MAS) of 96 Canadian watersheds based on the elasticity of streamflow in relation to precipitation, PET and human impacts such as land use and cover change. Elasticities of streamflow for each watershed are analytically derived using the Budyko Framework. We found that climate change generally caused an increase in MAS, while human impacts generally a decrease in MAS and such impact tends to become more severe with time, even though there are exceptions. Higher proportions of human contribution, compared to that of climate change contribution, resulted in generally decreased streamflow of Canada observed in recent decades. Furthermore, if without contributions from retreating glaciers to streamflow, human impact would have resulted in a more severe decrease in Canadian streamflow.

Regional changes in precipitation (*P*) minus evapotranspiration (*E*), *P* — *E*, caused by changes in specific humidity, circulation, and moisture transports complicate impacts of climate change on changes to streamflow^1^. Human activities such as river regulations^2^,^3^,^4^,^5^,^6^, land use change^7^,^8^,^9^, deforestation^10^, reforestation^11^, and extraction or diversion from surface water and groundwater^9^,^12^,^13^,^14^ generally incur a direct change to streamflow. Most studies attributed changes in streamflow to climate change impact only, such as a shift from snowfall towards rainfall in USA^15^, increasing trends in annual total precipitation in Canada^16^, without considering possible impact of human activities which can alter the streamflow directly through influencing the runoff production and indirectly through affecting the climatic variables^17^,^18^,^19^.

It is a challenge to quantify human contribution to changes in streamflow^20^ partly because human disturbances incur changes to many subsystems such as atmosphere, hydrosphere, cryosphere, land surface and biosphere^19^. Instead of using deterministic rainfall-runoff models to assess the impacts of climate change or human activities on runoff for each watershed, in this study we used observed precipitation, streamflow and estimated PET data to separate direct impacts of human activities from impacts of climate change (even though the latter is related to human impacts) to changes in Canadian streamflow based on the Budyko Framework^21^. The Budyko Framework assumes that the long-term water balance of a watershed based on the dryness index of actual evaporation to precipitation, *E*/*P*, is primarily a function of the atmospheric supply and demand of water, expressed as the ratio of PET to precipitation, *E*_*p*_/*P*, i.e., *E*/*P* =* f* (*E*_*p*_/*P*, *n*), where *n* is an empirical coefficient representing the combined watershed landscape properties that controls water-energy balances^22^ (Supplementary Fig. S1). In other words, the Budyko Framework enables us to predict hydrologic responses of a watershed to a wide range of climatic conditions with respect to the characteristics of the watershed, such as land properties, terrain features and others.

The long-term moving average water balance with a 20-year window, *P* = *E* + *R* (*R* is streamflow), of 96 Canadian watersheds (Supplementary Figs S2 and S3, Tables S1 and S2) was analyzed, but changes in water storages of watersheds are ignored in the analysis. The elasticity of streamflow, defined as the percentage change of streamflow from a 1% annual change in climate or land conditions and analytically derived from the Budyko Framework^23^, was used to estimate hydrological responses to climate change, land use and cover changes (LUCC) for each watershed. Hydroclimatic data was separated to two subseries for pre- and post-change periods (periods-1 and -2, respectively) from an abrupt change-point. Thus, the relative contribution of changes in precipitation and PET, and the relative contribution of human activities (LUCC) to streamflow change from periods-1 and -2, can be obtained from the difference in precipitation, PET and *n* between periods-1 and -2, and the corresponding streamflow change based on its elasticity to the above changes. The separate results were further validated by a decomposition method^24^ that are also analytically derived from the Budyko Framework. Details of the above processes are described in Methods and Supplementary Methods.

## Results

### Hydroclimatic trends and change-points

**Figure 1:**
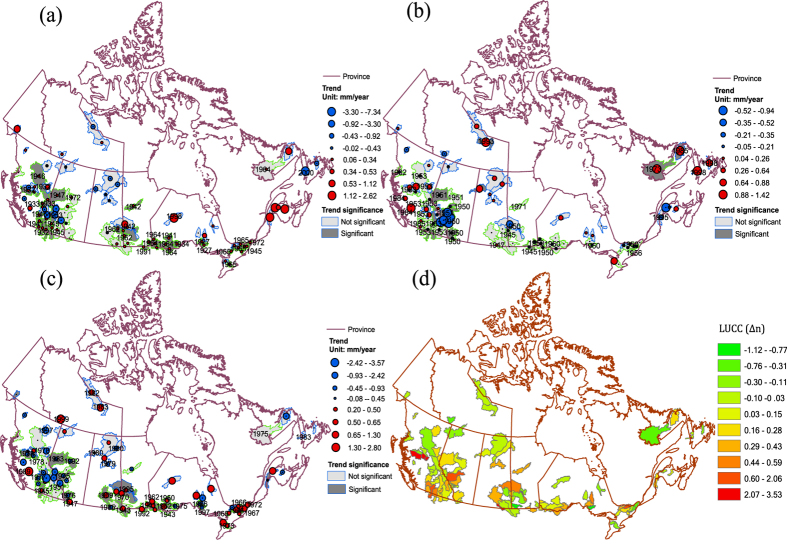
Change-points and trends of the annual precipitation (**a**), potential evaporation (**b**) and streamflow (**c**) in mm/year, and (d) change in landscape parameter *n* (d; Δ*n* = *n*_2 _− *n*_1_) of 96 selected watersheds across Canada. For Fig. 1a–c, only change-points (in year) that are statistically significant at 10% significant level are presented. Blue (green) boundaries show RHBN (non-RHBN) watersheds selected. Light (deep) grey watersheds represent trends that are not (are) statistically significant. The magnitudes of trends are presented in terms of circle sizes, in which green (red) circles represent decreasing (increasing) trends. Maps in Fig. 1 were generated with licensed ArcGIS 10.2 using public domain geographic data of the Atlas of Canada 1,000,000 National Frameworks Data (http://geogratis.gc.ca/).

Change-point and trend analysis (Methods) of 96 selected watersheds are shown in Fig. 1. Out of 60 non-RHBN (Reference Hydrometric Basin Network) watersheds, the streamflow and PET of 41 watersheds and the precipitation of 45 watersheds shows statistically significant change-points. However, for 36 RHBN watersheds, the corresponding number of watersheds decreases to 9 and 2 respectively, mainly because of pristine land-use conditions of RHBN watersheds. Change-points of streamflow data are mainly detected around 1929–1997 (mean 1969) and 1977–2004 (mean 1991) for non-RHBN and RHBN stations, respectively. Therefore, we adopt 1990 as the change point year for RHBN stations. However, for non-RHBN stations, because data begins no later than 1960, we adopt 1980 as the change point year to ensure sufficient length of data before and after change points. By adopting a common change point year for non-RHBN and RHBN stations, we have a better basis to compare the attribution results between different non-RHBN and RHBN watersheds.

From periods-1 to -2, the annual streamflow generally decreased (0–81 mm) along the Canadian Rockies (CR) which are Boreal and Montane Cordillera shown in Supplementary Fig. S2) but increased (0–58 mm) elsewhere, and 35 non-RHBN in mid- and eastern Canada, and 3 RHBN watersheds show statistically significant increasing trends between 1940 s-2010 (Fig. 1c). However, depending on the periods considered (30-, 40- and 50-year), some RHBN streamflow data in southern Canada showed significant decreasing trends^25^, but most showed insignificant decreasing trends between 1970–2010.

Change-points of annual precipitation (PET) occurred in about 1955 (1950) and 1992 (1989) for non-RHBN and RHBN watersheds, respectively. Annual precipitation has increased 5–35% in southern Canada over 1990–1998^26^, and abrupt changes often happened earlier in western than in eastern watersheds (Fig. 1a). For non-RHBN watersheds, differences in detected change-points between streamflow and climate data can be attributed to human activities. From periods-1 to -2, changes to the mean annual precipitation range from -44 to 158 mm, with a significant decrease in central CR and northern Canada but a significant increase in southern Canada. Most British Columbia (BC) and northern Canada showed an increase (0–30 mm) in the mean annual PET, but southern Canada generally a decrease (0–18 mm) (Fig. 1b), while *n* had generally increased in southern but some had decreased in northern watersheds of Canada (Fig. 1d).

### Elasticities of streamflow

For RHBN and non-RHBN watersheds, *n* ranges 0.442–3.295 and 0.285–9.305, with a mean value of 1.279 and 2.218, respectively (see Supplementary Tables S1 and S2). Higher *n* means higher *E* for a given *P* and *E*_*p*_, and hence a lower runoff (*R*). For example, non-RHBN watersheds #4 and #13 have similar *P* (about 1000 mm) and *E*_*p*_ (about 700 mm), but watershed #4 has high *R* (about 500 mm) because of low *n* (1.525) while watershed #13 has low *R* (about 350 mm) because of high *n* (2.712). From periods-1 to -2, most southern (northern) watersheds have become wetter (drier), as *E*_*p*_/*P* in the south (north) decreases (increases). *E*/*P* tends to increase especially in CR but it also decreases elsewhere (Supplementary Fig. S5). *E*/*P~E*_*p*_/*P* relationships for most watersheds in periods-1 and -2 do not follow the same Budyko curve (Supplementary Fig. S6), which likely implies that streamflow changes were induced by human impacts, especially when they change in an opposite manner.

**Figure 2:**
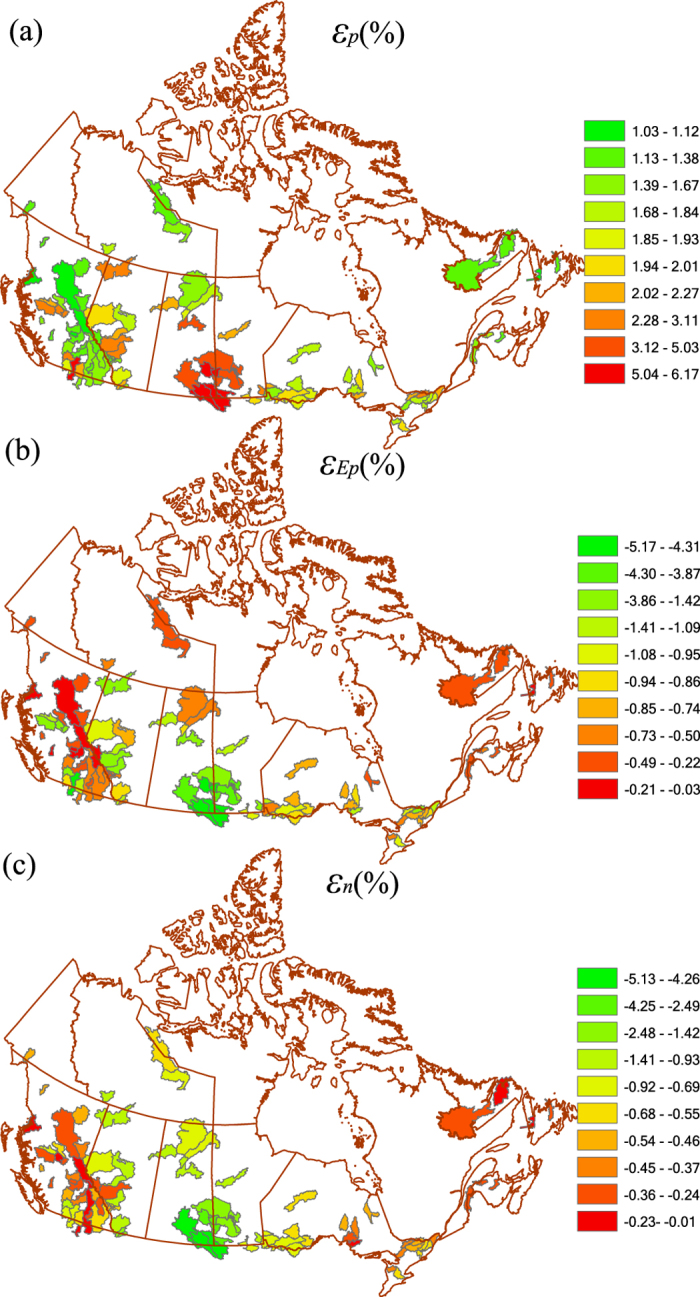
Elasticity of streamflow of 96 watersheds of Canada to (**a**) precipitation *ε*_*p*_, (**b**) potential evaporation (PET), *ε*_*Ep*_, and (**c**) the watershed landscape *ε*_*n*_. Maps in Fig. 2 were generated with licensed ArcGIS 10.2 using public domain geographic data of the Atlas of Canada 1,000,000 National Frameworks Data (http://geogratis.gc.ca/).

Figure 2 shows the spatial distribution of elasticity of streamflow to precipitation, *ε*_*p*_, to PET, *ε*_*Ep*_ and to LUCC, *ε*_*n*_ for Canada. The ranges of *ε*_*p*_ are 0.03–5.17, for *ε*_*Ep*_ are −5.17 −0.03, and for *ε*_*n*_ are −5.14 −0.02. The mean *ε*_*p*_, *ε*_*Ep*_ and *ε*_*n*_ values are 2.38, −1.38 and −1.03 for non-RHBN watersheds, and 1.65, −0.65 and 0.61 for RHBN watersheds, respectively. The spatial pattern of *ε*_*p*_ is somewhat similar to that of ecozone, and land use/cover (Supplementary Fig. S2 and S3). As expected, streamflow of the semi-arid Canadian Prairies (CP), which comprises of Alberta (AB), Saskatchewan (SK), and Manitoba (MB), is highly sensitive to LUCC, but less sensitive to LUCC in CR and northern Canada, e.g., absolute values of *ε*_*p*_, *ε*_*Ep*_ and *ε*_*n*_ of southern CP are higher than other parts of Canada (Fig. 2).

### Direct human impacts and climate change to streamflow change

**Figure 3:**
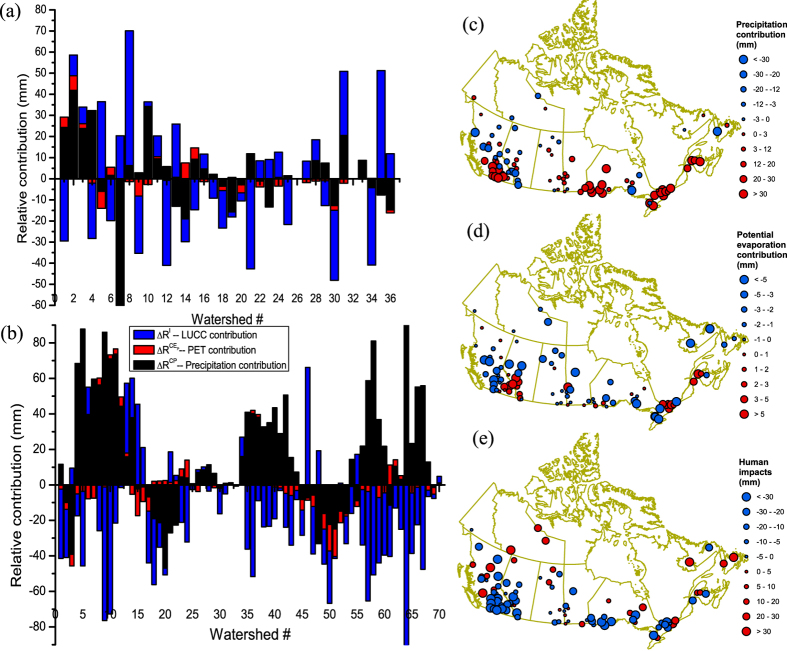
Relative contributions of changes in precipitation (**c**), PET (**d**) and landscape (**e**) to changes in the annual streamflow of selected Canadian RHBN (**a**) and non-RHBN (**b**) watersheds, represented by blue, red and black bars, respectively. Descriptions of watersheds of # shown in Fig. 3a are given in Supplementary Table S1 and in Fig. 3b are given in Supplementary Table S2, respectively. Maps in Fig. 3 were generated with licensed ArcGIS 10.2 using public domain geographic data of the Atlas of Canada 1,000,000 National Frameworks Data (http://geogratis.gc.ca/).

Streamflow change results from changes in precipitation, 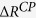, PET, 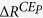, and LUCC, 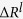 representing human impacts (Fig. 3). The modeled streamflow change 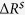
(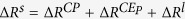) based on the elasticity method generally agrees well with the observed 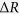
(Supplementary Fig. S7), with an average absolute error and a Pearson correlation of 4.3 mm and 0.98, respectively. Since precipitation and LUCC exerts opposite influence on streamflow, the net effect might lead to a minimal change in streamflow of watersheds subjected to both climate and LUCC impacts. However, their relative contributions to streamflow change can be deciphered, e.g., RHBN (#1, #4) and non-RHBN (#9, #10, #57 and #64) watersheds showed significant increase in streamflow (26–88 mm) due to increasing precipitation, but significant decrease in streamflow (28–87 mm) due to increasing *n*, which results in minimal streamflow changes. Therefore, human activities represented by LUCC tend to decrease the streamflow, as already observed in Canadian streamflow, albeit precipitation over Canada has generally increased.

In Canada, streamflow change is mainly controlled by changes in precipitation than PET (Fig. 3a,b). From periods-1 to -2, ranges of 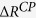, 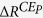 and 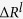
are [−140.5, 41.9], [−8.2, 7.5] and [−42.7, 63.8] mm for RHBN watersheds, and [−46.9, 137.4], [−15.4, 11.2] and [−123.6, 66.2] mm for non-RHBN watersheds, respectively. Their corresponding mean values are 2.4, −0.5 and −0.7 mm for RHBN watersheds, and 17.3, −1.9 and −14.8 mm for non-RHBN watersheds, respectively. As expected, human impacts have a significantly higher contribution to the decrease of streamflow in non-RHBN watersheds than RHBN watersheds. Spatial patterns of 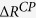, 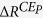 and 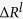 are shown in Fig. 3c–e, which are similar to trend analysis of precipitation, PET and *n* (Fig. 1). Precipitation (human impacts) generally contributed to an increase (decrease) in the streamflow of southern Canada, even though there are exceptions.

We also used the decomposition method^24^ based on Budyko Framework (Supplementary Fig. S1 and Methods) to validate the relative contribution of human activities and climate change to streamflow change. Although the former merely attributes streamflow change to climate change and direct human impacts, without considering the contribution of precipitation and PET separately, the overall results are similar to that of the elasticity method from comparing the results derived from the decomposition and elasticity methods (Supplementary Fig. S8).

## Discussion

The elasticity and decomposition methods built on the Budyko framework involve uncertainties, such as separating relative contributions of climate change and human impacts on changes to streamflow, abrupt change and temporal trends of streamflow. For example, assuming 1980 and 1990 to be the change-point year for non-RHBN and RHBN watersheds, respectively, and climatic regimes and human impacts remained relatively stable in both periods, etc., may be not true. Therefore, we further analyzed streamflow changes under five 10-year windows attributed to climate change and human impacts from 1961 to 2010, relative to the 1930–1960 base period, for 30 (mostly non-RHBN) watersheds.

**Figure 4:**
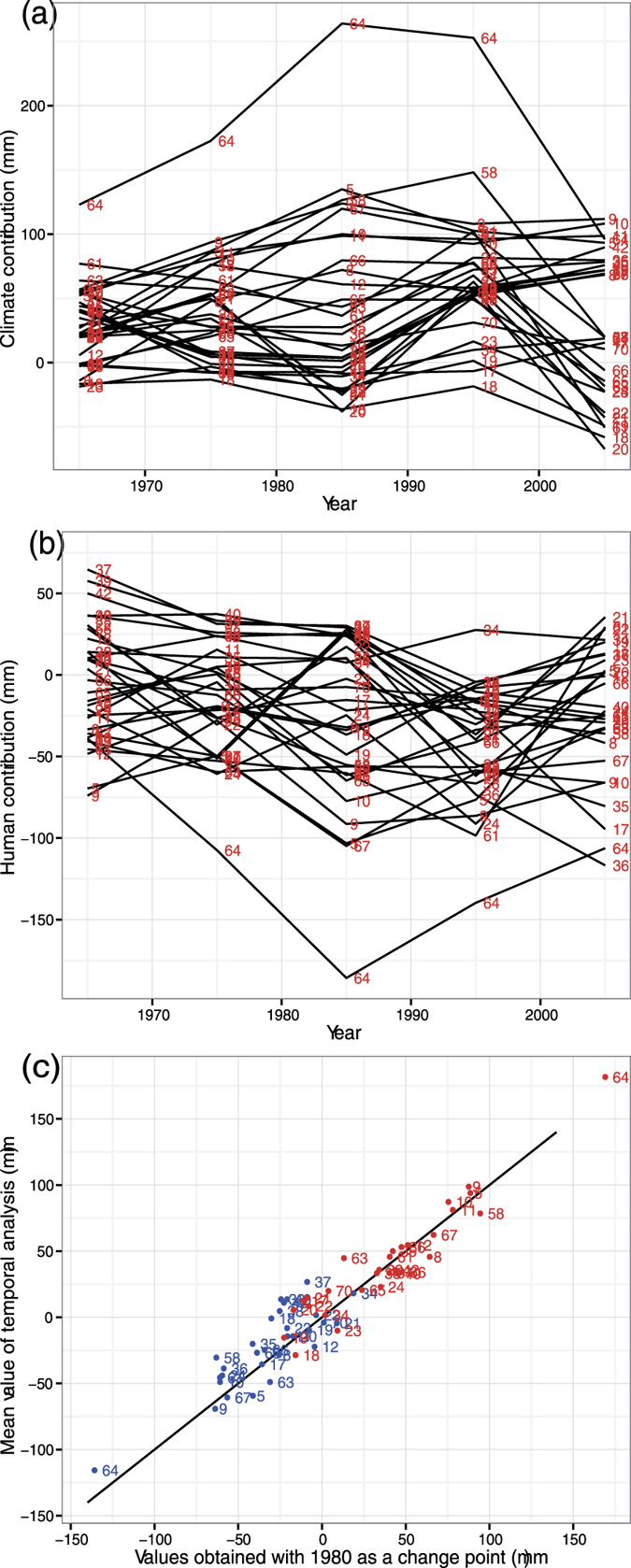
Temporal Budyko analysis results of Contributions of climate (**a**) and human (**b**) to changes in MAS from the baseline 1931–1960 period at 10 year intervals for 30 watersheds estimated from the decomposition method based on the Budyko framework; and scatterplots between changes in MAS due to contributions of climate (red dots) and human (blue dots) averaged over 5 10-year periods, and changes in MAS based on 1980 as the assumed change-point for each watershed. The watershed numbers shown in the figures are described in Supplementary Table S2.

From 1960s onward, human activities generally lead to decreasing streamflow until 2010 (Fig. 4a), while climate change predominantly lead to increasing streamflow but the impact could be opposite for some watersheds until about 1980s, when the reverse happened (Fig. 4b). The range of standard deviations (mean) in climate and human contributions to streamflow change over the five 10-year windows of data analyzed for the 30 watersheds was 8.5–75.3 mm (33.7 mm) and 8.8–54.8 mm (29.4 mm), respectively. It seems that the mean contributions of climate change and human activities to streamflow change of these 30 watersheds obtained from the five 10-year window analysis for 1961–2010 agree well with results obtained from using 1980 as the common change-point for the 30 watersheds (Fig. 4c).

Human impacts on streamflow change of the 96 watersheds divided into 10 groups were further explored using a correlation analysis between streamflow change and human activities such as LUCC, increased municipal water consumption due to population increase and increased evaporation due to water impounded behind dams. In each group all watersheds have comparable range of streamflow change and human activities categorized under population density, number of dams, percentage of cropland and rangeland.

Because of a lack of long-term data related to human activities, we assume that streamflow to landscape changes detected for each watershed to be directly dependent on certain indicators collected in a particular year only. The relationships between “human” indicators and streamflow change for these 10 groups of watersheds are shown in Supplementary Fig. S9. The proportion of impervious areas of urban watersheds with large population density tends to grow over the years, resulting in decreased infiltration but increased surface runoff. Conversely, water impoundment by dams results in increased evaporation loss and so decreased streamflow. The expansion of cropland means converting perennial vegetation to seasonal cropping systems that reduces annual evapotranspiration and increased streamflow during non-growing season. On the other hand, rangelands could have higher evapotranspiration than natural lands, resulting in less streamflow. However, streamflow could change in a manner opposite to above relationships, e.g., irrigated lands could have higher evapotranspiration than natural lands which resulted in less streamflow. Further, crop water consumption depends on crop types, and so streamflow could decrease with intensive cultivation of certain crop types. Therefore, human impacts on the streamflow change could depend on various combinations of physical and climatic factors.

In addition to the above human indicators, we have also considered trends of normalized difference vegetation index (NDVI), which is related to the percent of green cover, as a possible factor contributing to decreased streamflow in Canada. Supplementary Fig. S9e shows the negative correlation between NDVI trends for the first half of August over 1981–2011 and annual streamflow change of Canada. As most Canada landmass became greener (NDVI increase) in southern and Arctic regions (Supplementary Fig. S11a) over 1981–2011, annual evapotranspiration could increase because of increased NDVI even though there is no consistently positive trend detected in the evapotranspiration of Canada^7^,^27^,^28^,^29^. Even though increased NDVI could be related to both the climate change and human contribution^30^, it is difficult to separate their relative contributions to the increased NDVI.

In high-latitude and mountainous regions of Canada, the widespread retreat of glaciers has contributed to increase in streamflow^31^,^32^,^33^,^34^. Since this study does not account for impacts of climate change on retreat of glaciers, it underestimated the actual human contribution to decreased streamflow. Some studies show that potential impacts of a warmer climate do not significantly affect the availability of water in snow-dominated regions such as Canada^17^,^35^. However, recent studies reported conflicting results on the sensitivity of streamflow to global warming impact, e.g., a general decrease in observed streamflow caused by a shift from snowfall to rainfall in USA^15^; and a projected increase (decrease) in streamflow of Canada (USA) under climatic change impact^36^ which may be partly because hydrologic impacts of human activities are not considered in hydroclimatic models. More detailed analysis will be necessary to better estimate anthropogenic impacts such as landuse changes and streamflow regulations to watersheds studied.

A limitation of Budyko-based methods for separating the relative contribution of human impacts and effects of climate change to the streamflow change in snow-dominated watersheds is that the change in snow ratio (SR, amount of snowfall to total precipitation) which is more related to the effect of climate change than direct human impact. Watersheds with a higher SR tend to have a lower *E*/*P* (Supplementary Fig. S10). This means that under similar climatic conditions and landscape properties, streamflow will tend to be higher. This empirical relationship has been found in watersheds of USA^15^ and China^37^, but its mechanism is still unknown. The stationary assumption of SR in Budyko-based methods is violated in some watersheds given North American Regional Reanalysis (NARR) data show that SR has increased in southern Canada because of the increase in winter precipitation, but SR has decreased in northern Canada over 1979–2014 (Supplementary Fig. S11b), as also been observed in station climate data^26^. Given that streamflow in southern Canada has decreased even though it should have increased because of increased SR, the contribution of human impact to decreased streamflow could have been higher.

## Methods

From analyzing the 1961–2010 annual water balance of 370 watersheds using streamflow data of Water Survey Canada (WSC), Wang, *et al.*^38^ found that large spatial variabilities of basin-scale water budget over Canada, and some significant discrepancies in the water budget of some watersheds in northern Canada (above 60°N) were partly due to mass loss of glaciers. Given the Budyko framework is meant for the long-term (>1 year) water balance analysis, 96 Canadian watersheds with drainage area >2,000 km^2^ and an annual water imbalance <10% of the annual precipitation were selected for this study.

Since available streamflow data is less complete than precipitation and temperature data, only watersheds with long-term streamflow data were selected in this study. The RHBN streamflow data of WSC have been extensively used for climate change studies, since RHBN data are characterized by relatively pristine and stable land-use conditions (<5% of the land surface modified) with at least 20 years of record. A total of 36 RHBN watersheds with daily streamflow data of 1971–2010 were selected for this study. Further, daily streamflow data of 60 non-RHBN stations that began no later than 1960 were also selected for this study (Supplementary Fig. S2, Tables S1 and S2). In this study, the total annual depth of streamflow was estimated for station drainage areas while the total annual values of other hydroclimatic variables were estimated for actual watershed areas. Next, the gridded, monthly precipitation dataset^39^, and the monthly PET dataset of CRU TS v. 3.22^40^ were also used under the Budyko framework. To estimate the change in SR over watersheds, we used the NARR^41^ snowfall and precipitation data over 1979–2014. Abrupt changes in the mean hydroclimatic data due to climatic changes and/or anthropogenic effects were detected using the nonparameteric Pettitt test^42^, monotonic trends was investigated by the Mann-Kendall (MK) test^43^, and magnitudes of trend were estimated using the Theil–Sen approach^44^, at 10% significance level.

Various significant hydroclimatic change-points for Canada, mainly between 1970–1990, have been detected^45^. We first divided RHBN (non-RHBN) streamflow datasets into pre-1990 (pre-1980) and post-1990 (post-1980) parts, respectively. Hydroclimatic changes from periods-1 to -2 were estimated for the 96 watersheds. The contributions of human impacts to observed changes in MAS of Canada were assessed in terms of population density, dam distribution, and land uses (Supplementary Fig. S3) obtained from Natural Resources Canada (www.geogratis.cgdi.gc.ca/geogratis/DownloadDirectory?lang=en), and remotely sensed, 8-km resolution, NDVI data of the first half of August for 1981–2011 obtained from the Global Inventory Modeling and Mapping Studies (GIMMS)^46^.

The Budyko framework simplifies the water-energy balance of large watersheds (>1,000 km^2^) over long time periods (>1 year) by apportioning precipitation to actual evaporation (*E*) and streamflow (*R*). Since PET (*E*_*P*_) and precipitation (*P*) are measures of energy and water available, respectively, the Budyko framework, *E*/*P *=* f* (*E*_*p*_/*P*, *n*), represents the water balance of a watershed in a stationary condition (Supplementary Figs S1, S4 and S6). Various climatic conditions represented by *E*_*p*_/*P* fall on a Budyko curve that only depends on watershed properties represented by one or more parameters. In this study, the Budyko curve^47^, Equation (1), was chosen because it is only described by one parameter *n* that is an empirical coefficient representing combined watershed landscape properties. A larger *n* value means more evaporation is partitioned from the precipitation and vice versa.


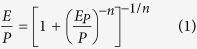


The long-term moving average *E* with a 10-year window is derived by water balance, *P* = *R* + *E*, where variations of water storages were neglected:


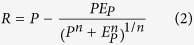


Next, *ε*_*p*_, *ε*_*Ep*_, and *ε*_*n*_ can be analytically derived and estimated from long-term *P*, *E*_*P*_ and *n* data. Assuming *P*, *E*_*P*_ and *n* are independent variables, thus Equation (2) can be interpreted as 
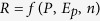
 with the total differential, *dR*:


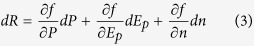


The elasticity of streamflow to, precipitation, PET and watershed landscape can be defined as 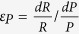, 
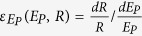
 and 
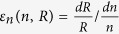
, respectively. Equation (3) can be rearranged as:


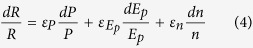



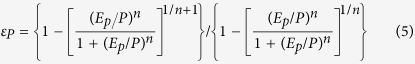



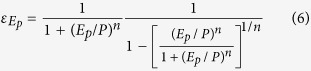



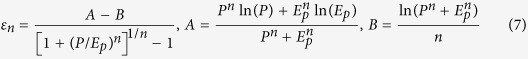


Using Equations (5, 6, 7), the elasticity of streamflow for a watershed can be estimated from its long-term data of *P*, *E*_*P*_ and *n*.

Supplementary Fig. S4 illustrates the relationship between *ε*_*p*_ and *E*_*p*_/*P* for selected watersheds of Canada. As expected, *ε*_*p*_ increases with *E*_*p*_/*P*, but, *ε*_*Ep*_ decreases with *E*_*p*_/*P* because streamflow increases with precipitation but decreases with PET. The elasticity of streamflow is less dependent on *E*_*p*_/*P* when climate is dry (*E*_*p*_/*P* > 1.5), but less dependent on *n* when climate is extremely wet (*E*_*p*_/*P* < 0.5). *ε*_*p*_ is close to 1.0 but *ε*_*Ep*_ is close to 0.0 under extremely humid climate, and are almost independent of landscape conditions. On the other hand, *n* tends to affect the climate elasticity when *E*_*p*_/*P* is between 0.5 and 1.5. Changes of the mean annual runoff of a watershed from period-1 (*R*_1_) to period-2 (*R*_2_), Δ*R* = *R*_2_ − *R*_1_, could be due to the combined effect of climate change Δ*R*^*C*^, and the watershed LUCC Δ*R*^*L*^, i.e., Δ*R *=* *Δ*R*^*C*^ + Δ*R*^*l*^, where Δ*R*^*C*^ = Δ*R*^*CP*^ + Δ*R*^*CEP*^, and Δ*R*^*CP*^ and Δ*R*^*CEP*^ are the streamflow change caused by changes in precipitation and PET, respectively. Therefore, 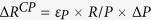, 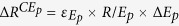 and 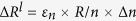, where Δ*P *=* P*_2_ − *P*_1_, Δ*E*_*P*_* *=* E*_*P*2_ − *E*_*P*1_ and Δ*n *=* n*_2_ − *n*_1_, respectively.

Since the elasticity method uses only a first-order approximation of streamflow change in Equation (4), an error analysis was conducted to test the validity of the elasticity method. Following Yang, *et al.*^48^, Equation (2) was expanded by Taylor’s series to estimate errors associated with using a first-order approximation for estimating streamflow change. The results show that in 94 out of 96 watersheds, the relative error of approximating precipitation change to the streamflow change is less than 9%. Therefore, it is acceptable to apply the elasticity method in this study.

The decomposition method^24^ (Supplementary Methods) offers another explanation to streamflow responding to effects of climate change and human activities based on the Budyko framework. This method also assumes no indirect human-induced streamflow change resulted from human influence on the climate change. Unlike the elasticity method which uses a first-order approximation of the Budyko equation (Equation 3), the decomposition method considers that changes of *E*_*p*_/*P* of a watershed along the horizontal direction in its Budyko curve only result from climate change impact, while changes of *E*/*P* along the vertical direction in its Budyko curve result from both climate change and direct human impacts^24^ (Supplementary Fig. S1). The streamflow change is divided into two parts, so that Budyko-based methods can separately account for direct human-induced and climate-induced streamflow changes due to changes in both precipitation and PET.

## Additional Information

**How to cite this article**: Tan, X. and Gan, T. Y. Contribution of human and climate change impacts to changes in streamflow of Canada. *Sci. Rep.* **5**, 17767; doi: 10.1038/srep17767 (2015).

## Supplementary Material

Supplementary Information
