# Supplementary material for: Contribution of human and climate change impacts to changes in streamflow of Canada
Source: Sci Rep. 2015 Dec 4;5:17767. doi: 10.1038/srep17767 (PMC4669504; doi:10.1038/srep17767)
Supplement: Supplementary Information [file srep17767-s1.pdf]

Supplementary Information

Contribution of human and climate change impacts to changes in streamflow of Canada

Xuezhi Tan[1,2], Thian Yew Gan[1,*]

[1]Department of Civil and Environmental Engineering, University of Alberta, Edmonton, Alberta, Canada

[2]State Key Laboratory of Water Resources and Hydropower Engineering Science, Wuhan University, Wuhan, China

* Corresponding author, tgan@ualberta.ca

The Supplementary Information included in our paper comprises:

1. Supplementary Methods.
2. Supplementary Table S1 and S2
3. Supplementary Figure S1-S11.

Supplementary Methods

Decomposition method

Because of both climate change and direct human interferences, a watershed could shift over time from points A at period-1 (E_{p1}/P_1) to B at period-2 (E_{p2}/P_2) in Supplementary Fig. S1. However, based on the Budyko hypothesis, the watershed should evolve from A to C along the Budyko curve that represents the impact of climate change only. Given same climatic conditions between B and C but without impacts of human activities at C, the precipitation, evaporation, dryness and evaporation indices of C are also P_2, E'_2, E_{p2}/P_2 and E'_2/P_2, respectively. Thus, the horizontal shift from E_{p1}/P_1 to E_{p2}/P_2 is due to the climate change, while the vertical shift from E_1/P_1 to E_2/P_2 is due to both climate change and direct human impacts. So the vertical shift can be decomposed to climate change effects from E_1/P_1 to E'_2/P_2 and direct human activity effects from E'_2/P_2 to E_2/P_2. This method first calibrates the Budyko curve to each watershed with data of period-1 to obtain n without the effect of human activities, and then the evaporation ratio of period-2 due to climate change only is E'_2/P_2, where the observed dryness index is E_{p2}/P_2. Thus, the streamflow change caused by direct human impacts is $\Delta R^H = \Delta P - \Delta E = P_2(E'_2/P_2 - E_2/P_2)$ while that by climate change is $\Delta R^C = \Delta R - \Delta R^H = P_2(1 - E'_2/P_2) - R_1$.

Supplementary Table S1 List of the 36 hydrometric stations/watersheds and their mean annual water budget estimates during the period-1 and period-2

No.: Hydrometric station number; ***A***: Drainage area (km^2); ***R***: Streamflow (mm); ***E***: Evapotranspiration (mm); ***P***: Precipitation (mm); $\boldsymbol{E_p}$: Potential evapotranspiration (mm); 1,2: period-1 and period-2; **Δ:** the difference of water budget component between the period-2 and period-1 (e.g., **Δ*R*= *R*2-*R*1**); *n*, watershed landscape parameter; *Y*, year of streamflow abrupt change point. All water variables are mean annual values starting in 1971 and ending in 2010 in mm $year^{-1}$.

No.	ID	Station Name	Location (N,W)		*A*	*R*1	*R*2	Δ*Q*	*E*1	*E*2	Δ*E*	*P*1	*P*2	Δ*P*	E_p1	E_p2	ΔE_p	*n*	*Y*
1	01AD002	Saint John River at Fort Kent	47.3	-68.6	14700	627	625	-2	451	481	31	1077	1106	28	636	626	-10	1.214	2002
2	01BC001	Restigouche River below Kedgwick River	47.7	-67.5	3160	667	720	53	422	417	-5	1089	1137	48	602	587	-15	1.148	2002
3	01BE001	Upsalquitch River at Upsalquitch	47.8	-66.9	2270	560	586	26	528	529	1	1088	1115	27	613	609	-4	1.930	2004
4	01BO001	Southwest Miramichi River at Blackville	46.7	-65.8	5050	750	762	12	410	434	25	1159	1196	37	660	665	5	0.968	1984
5	02FC001	Saugeen River near Port Elgin	44.5	-81.3	3960	474	501	27	542	508	-34	1017	1009	-7	677	693	16	1.754	1999
6	02KB001	Petawawa River near Petawawa	45.9	-77.3	4120	393	378	-15	570	587	17	963	965	2	670	664	-6	2.263	1985
7	02YL001	Upper Humber River near Reidville	49.2	-57.4	2110	1226	1181	-46	93	5	-88	1319	1185	-134	567	582	15	0.459	1983
8	02YQ001	Gander River above Falls	49.0	-54.9	4450	835	912	77	382	312	-70	1217	1224	7	580	583	4	0.951	1990
9	03QC001	Eagle River above Falls	53.5	-57.5	10900	753	716	-37	331	371	40	1084	1087	3	433	448	14	1.108	1985
10	04DA001	Pipestone River at Karl Lake	52.6	-90.2	5960	286	318	32	394	406	13	680	724	44	554	561	6	1.616	1998
11	04JC002	Nagagami River at Highway No.11	49.8	-84.5	2410	312	338	26	427	413	-14	738	751	12	584	582	-2	1.606	1991
12	04LJ001	Missinaibi River at Mattice	49.6	-83.3	8940	355	321	-34	463	504	41	818	825	7	583	585	1	1.807	1997
13	04MF001	North French River near the Mouth	51.1	-80.8	6680	436	445	8	354	329	-24	790	774	-16	578	576	-2	1.113	1987
14	05BB001	Bow River at Banff	51.2	-115.6	2209.6	550	520	-30	265	272	7	815	792	-23	533	506	-27	0.738	2000
15	05LH005	Waterhen River near Waterhen	51.8	-99.5	55100	45	46	1	427	452	25	472	498	26	682	652	-29	3.009	2004
16	06BD001	Haul Tain River above Nobert River	56.2	-106.6	3680	152	162	10	324	321	-2	476	484	8	572	578	6	1.452	1999
17	06CD002	Churchill River above Otter Rapids	55.6	-104.7	119000	77	71	-7	415	426	11	493	497	4	584	584	-1	2.877	1979
18	06DA004	Geikie River below Wheeler River	57.6	-104.2	7730	200	177	-23	272	290	18	472	466	-5	505	513	8	1.172	1980
19	07AA002	Athabasca River near Jasper	52.9	-118.1	3872.7	700	688	-12	224	218	-6	924	907	-18	494	492	-2	0.602	1999
20	07CD001	Clearwater River at Draper	56.7	-111.3	30791.6	124	114	-9	324	329	4	448	443	-5	558	574	16	1.634	1980
21	07FB001	Pine River at East Pine	55.7	-121.2	12100	499	475	-24	249	287	38	748	762	13	523	521	-2	0.737	1977
22	07LE002	Fond Du Lac River at Outlet of Black Lake	59.1	-105.5	50700	187	192	5	262	255	-7	449	448	-2	504	516	12	1.152	1983
23	07OB001	Hay River near Hay River	60.7	-115.9	51700	76	73	-3	330	306	-24	406	379	-27	534	536	2	2.151	2004
24	07RD001	Lockhart River at Outlet of Artillery Lake	62.9	-108.5	26600	139	151	12	144	133	-11	283	284	1	366	388	22	0.846	1983
25	08CD001	Tuya River near Telegraph Creek	58.1	-130.8	3550	334	326	-9	171	181	10	506	507	1	384	383	-2	0.717	1981
26	08FB006	Atnarko River near the Mouth	52.4	-126.0	2550	354	331	-23	549	576	28	902	907	5	453	464	11	--	1997
27	08JB002	Stellako River at Glenannan	54.0	-125.0	3600	166	175	9	372	363	-9	538	537	0	506	511	4	2.027	1979
28	08JE001	Stuart River near Fort St.James	54.4	-124.3	14200	279	300	21	251	241	-10	529	540	11	547	552	5	0.884	1995
29	08LA001	Clearwater River near Clearwater Station	51.6	-120.1	10300	675	687	12	196	193	-3	872	880	8	564	568	5	0.529	2002
30	08LD001	Adams River near Squilax	50.9	-119.7	3210	724	675	-48	334	368	33	1058	1043	-15	583	590	7	0.776	1984

31	08MA002	Chilko River at Outlet of Chilko Lake	51.6	-124.1	2310	544	617	74	199	149	-51	743	766	23	496	507	11	0.585	1989
32	08NB005	Columbia River at Donald	51.5	-117.2	9700	555	529	-26	808	785	-23	1363	1314	-48	492	491	-2	--	2000
33	09AC001	Takhini River neat Whitehorse	60.9	-135.7	7050	279	280	1	146	156	10	425	436	11	397	398	1	0.651	1994
34	10CD001	Muskwa River near Fort Nelson	58.8	-122.7	20300	359	320	-39	182	216	33	541	536	-5	429	428	-1	0.691	1997
35	10FA002	Trout River at Highway No.1	61.1	-119.8	9270	117	163	46	266	209	-57	383	372	-11	503	506	3	1.398	1999
36	10PB001	Coppermine River at Outlet of Point	65.4	-114.0	19200	171	183	12	95	65	-30	266	249	-17	338	350	12	0.591	1982

Supplementary Table S2 List of the 60 non-RHBN and 10 RHBN hydrometric stations/watersheds and their mean annual water budget estimates during the period-1 and period-2

No.1: Hydrometric station number including both non-RHBN and RHBN; **No.2:** Hydrometric station number including only non-RHBN; ***A***: Drainage area (km^2); **Year**: The initial year of the available annual streamflow data; ***R***: Streamflow (mm); ***E***: Evapotranspiration (mm); ***P***: Precipitation (mm); E_p: Potential evapotranspiration (mm); 1,2: period-1 and period-2; **Δ:** the difference of water budget component between the period-2 and period-1 (e.g., **Δ*R*= *R*2-*R*1**); *n*, watershed landscape parameter; *Y*, year of streamflow abrupt change point. All water variables are mean annual values ending in 2010 in mm $year^{-1}$.

No.1	No.2	ID	Station Name	Location (N,W)		*A*	Year	*R*1	*R*2	Δ*Q*	*E*1	*E*2	Δ*E*	*P*1	*P*2	Δ*P*	E_p1	E_p2	ΔE_p	*n*	*Y*
1	1	02AB006	Kaminisiquia River at Kaministiquia	48.5	-89.6	6475	1935	302	270	-31	457	505	47	759	775	16	613	618	5	1.686	1975
2	2	02BD002	Michipicoten River at High Falls	48.0	-84.9	5130	1934	440	391	-49	482	518	37	922	910	-12	572	578	7	1.832	1986
3	3	02BE002	Montreal River near Montreal River Harbour	47.2	-84.6	2880	1936	458	415	-43	636	635	-1	1095	1050	-44	606	614	8	4.946	1997
4	4	02EB006	Muskoka River below Bala	45.0	-79.7	4770	1938	490	542	51	504	535	31	994	1077	82	679	690	10	1.525	1965
5	5	02FC001	Saugeen River near Port Elgin	44.5	-81.3	3960	1930	444	491	47	469	532	63	913	1024	110	684	692	8	1.350	1965
6	6	02GB001	Grand River at Brantford	43.1	-80.3	5210	1948	332	378	46	548	554	6	880	932	52	714	731	17	1.959	1973
7	7	02GE003	Thames River at Thamesville	42.5	-82.0	4300	1956	359	412	53	527	553	26	886	965	79	766	784	18	1.591	1971
8	8	02HL001	Moira River near Foxboro	44.3	-77.4	2620	1930	349	388	39	500	536	36	849	924	75	710	701	-9	1.573	1972
9		02KB001	Petawawa River near Petawawa	45.9	-77.3	4120	1930	354	378	24	512	598	86	866	976	110	668	670	2	1.813	
10	9	02KC009	Bonnechere River near Castleford	45.5	-76.6	2380	1930	246	261	15	547	629	82	793	890	96	679	675	-4	2.412	1966
11	10	02KD004	Madawaska River at Palmer Rapids	45.3	-77.5	5800	1930	361	418	58	496	533	37	856	951	95	686	679	-7	1.624	1966
12	11	02KF006	Mississippi River at Appleton	45.2	-76.1	2900	1930	325	372	47	503	515	13	828	887	60	716	703	-13	1.632	1967
13	12	02LA004	Rideau River at Ottawa	45.4	-75.7	3830	1949	309	365	56	553	518	-35	862	884	21	718	714	-5	2.007	1969
14	13	02LB005	South Nation River near Plantagenet Springs	45.5	-75.0	3810	1950	342	392	50	634	632	-2	976	1024	48	707	716	9	2.712	1972
15	14	03OE001	Churchill River above Upper Muskrat Falls	53.2	-60.8	92500	1954	584	600	16	350	332	-18	934	932	-3	437	467	29	1.359	1975
16		04JC002	Nagagami River at Highway No.11	49.8	-84.5	2410	1951	318	331	13	425	410	-15	743	741	-2	565	584	19	1.679	
17		04LJ001	Missinaibi River at Mattice	49.6	-83.3	8940	1930	378	331	-47	452	490	38	830	821	-8	571	587	15	1.734	
18	15	05AD007	Oldman River near Lethbridge	49.7	-122.9	17031	1913	160	114	-46	362	372	11	522	487	-35	841	828	-14	1.204	1976
19	16	05AE006	St.Mary River near Lethbridge	49.6	-112.8	3527	1930	128	97	-30	382	369	-13	510	466	-43	821	807	-14	1.456	1957
20		05BB001	Bow River at Banff	51.2	-115.6	2210	1930	562	528	-34	276	254	-22	838	782	-56	524	515	-10	0.715	
21	17	05BE004	Bow River near Seebe	51.1	-115.0	5170	1930	486	486	0	256	224	-32	743	710	-32	573	566	-7	0.696	1946
22	18	05BH004	Bow River at Calgary	51.1	-114.1	7868	1930	353	342	-11	369	350	-19	723	693	-30	599	592	-7	1.139	1946
23	19	05CC002	Red Deer River at Red Deer	52.3	-113.8	11609	1913	132	120	-12	439	458	20	570	578	8	664	648	-16	2.084	1929
24	20	05DF001	North Saskatchewan River at Edmonton	53.5	-113.5	28096	1912	242	218	-25	532	562	30	774	779	5	553	539	-15	3.479	1975
25	21	05JK002	Qu'Appelle River below Craven Dam	50.7	-104.8	32900	1955	5	5	0	356	365	10	361	371	10	854	872	18	3.911	1969
26	22	05LM001	Fairford River near Fairford	51.6	-98.7	79800	1956	30	32	3	442	462	19	472	494	22	631	651	20	4.560	1995
27	23	05MD004	Assiniboine River at Kamsack	51.6	-101.9	13000	1957	14	19	5	423	459	36	436	477	41	696	698	2	5.234	1995
28	24	05MD005	Shell River near Inglis	51.0	-101.3	4970	1957	17	23	6	426	467	41	443	490	47	694	696	3	4.813	1970

29	25	05ME001	Assiniboine River near Russell	50.8	-101.4	19400	1951	21	22	0	421	446	25	443	468	25	715	728	14	4.331	1994
30	26	05NB001	Long Creek near Estevan	49.1	-103.0	4840	1960	9	1	-8	394	381	-13	403	382	-21	862	875	13	4.379	1985
31	27	05ND007	Souris River near Sherwood	49.0	-102.0	23100	1931	5	3	-2	392	407	15	398	410	13	849	851	1	5.082	1942
32	28	05NF012	Souris River near Westhope	49.0	-101.0	43700	1931	5	6	0	388	399	11	394	405	11	871	870	-1	4.665	1943
33	29	05NG001	Souris River at Wawanesa	49.6	-99.7	61100	1954	8	7	-2	397	398	1	406	405	0	859	870	10	4.389	1995
34	30	05OC004	Pembina River at Neche	49.0	-97.6	8480	1920	21	42	21	487	520	33	508	562	54	746	743	-4	5.156	1992
35	31	05PA006	Namakan River at Outlet of Lac La Croix	48.4	-92.2	13400	1923	254	254	1	459	517	58	713	772	59	663	664	1	1.717	1943
36	32	05PA012	Basswood River near Winton	48.1	-91.7	4510	1926	272	261	-11	432	501	69	703	762	58	673	668	-5	1.461	1972
37	33	05PB014	Turtle River near Mine Centre	48.9	-92.7	4870	1921	241	272	31	460	484	23	702	756	54	655	651	-4	1.796	1962
38	34	05PC018	Rainy River at Manitou Rapids	48.6	-93.9	50200	1929	227	235	7	435	476	41	663	711	48	654	655	1	1.677	1941
39	35	05PC019	Rainy River at Fort France	48.6	-93.4	38600	1929	296	305	10	367	405	39	663	711	48	654	655	1	1.191	1941
40	36	05PE020	Winnepeg River below Lake of the Woods Outlets	49.8	-94.5	70400	1910	183	207	24	458	500	42	640	707	66	632	635	3	2.084	1962
41	37	05PH003	Whitemouth River near Whitemouth	49.9	-96.0	3750	1957	110	135	24	445	472	27	555	607	51	661	667	6	2.399	1996
42	38	05QA002	English River at Umfreville	48.9	-91.5	6230	1922	289	310	21	397	445	47	686	755	68	611	624	13	1.445	1950
43	39	05TD001	Grass River above standing stone Falls	55.7	-97.0	15400	1922	147	127	-20	331	377	46	477	504	26	557	579	22	1.569	1987
44	40	07BB002	Pembina River near Entwistle	53.6	-115.0	4402	1960	146	142	-3	393	408	16	538	551	12	631	642	12	1.808	1964
45	41	07BE001	Athabasca River at Athabasca	54.7	-113.3	74602	1955	191	165	-26	301	314	13	492	479	-13	580	606	26	1.192	1992
46	42	07EF001	Peace River at Hudson Hope	56.0	-121.9	73100	1952	451	505	54	118	56	-62	568	561	-8	453	469	16	0.464	1984
47	43	07GJ001	Smoky River at Watino	55.7	-117.6	50300	1958	232	192	-39	375	407	31	607	599	-8	566	584	18	1.554	1983
48	44	08DB001	Nass River above Shumal Creek	55.3	-129.1	18400	1956	1339	1323	-16	86	68	-18	1425	1392	-33	448	456	8	0.305	1968
49		08JB002	Stellako River at Glenannan	54.0	-125.0	3600	1959	202	161	-41	360	377	17	562	538	-23	486	507	22	1.819	
50	45	08JC001	Nechako River at Vanderhoof	54.0	-124.0	25200	1951	215	135	-81	466	519	53	681	654	-28	500	521	21	3.462	1978
51	46	08JC002	Nechako River at Isle Pierre	54.0	-123.2	42700	1956	230	179	-51	533	552	18	763	730	-33	470	490	21	7.214	1978
52		08JE001	Stuart River near Fort St.James	54.4	-124.3	14200	1951	304	286	-18	260	259	-2	564	545	-19	502	528	26	0.942	
53	47	08KB001	Fraser River at Shelly	54.0	-122.6	32400	1951	803	771	-32	54	69	15	857	840	-17	495	517	23	0.268	1977
54	48	08KH001	Quesnel River at Likely	52.6	-121.6	5970	1939	684	685	1	157	172	15	841	857	16	570	576	6	0.455	1957
55	49	08KH006	Quesnel River near Quesnel	52.8	-122.2	11500	1946	646	659	12	268	246	-22	915	905	-10	532	544	12	0.710	1959
56		08LA001	Clearwater River near Clearwater Station	51.6	-120.1	10300	1952	693	683	-10	163	196	34	855	880	24	552	568	16	0.465	
57		08LD001	Adams River near Squilax	50.9	-119.7	3210	1949	704	697	-8	282	358	76	986	1055	68	576	589	12	0.672	
58	50	08LE031	South Thompson River at Chase	50.8	-119.7	15800	1915	579	610	31	239	303	64	818	913	95	631	634	2	0.607	1954
59	51	08LF051	Thompson River near Spences Bridge	50.4	-121.4	55400	1952	442	436	-6	375	427	52	817	863	46	586	605	18	1.103	1977
60	52	08LG006	Nicola River near Spences Bridge	50.3	-121.2	7100	1958	126	108	-18	385	443	59	510	551	41	628	645	17	1.838	1977
61	53	08NA002	Columbia River at Nicholson	51.2	-116.9	6660	1917	512	492	-21	480	486	7	992	978	-14	522	503	-18	1.685	1977
62	54	08NE049	Colmbia River at Birchbank	49.2	-117.7	87400	1938	726	722	-4	508	524	16	1234	1246	12	527	522	-5	1.721	1946
63	55	08NH021	Kootenai River at Porthill	49.0	-116.5	34300	1929	416	398	-18	390	412	22	806	810	4	665	658	-7	1.077	1946
64	56	08NJ013	Slocan River near Crescent Valley	49.5	-117.6	3330	1926	829	862	33	345	470	125	1174	1332	158	586	585	-1	0.764	1951

65	57	08NL022	Similkameen River near Nighthawk	49.0	-119.6	9190	1929	228	213	-15	402	452	50	629	664	35	629	644	14	1.508	1946
66	58	08NM002	Okanagan River at Okanagan Falls	49.3	-119.6	6720	1917	71	84	13	572	651	79	643	736	93	663	666	3	5.829	1945
67	59	08NN012	Kettle River near Laurier	49.0	-118.2	9930	1930	257	267	10	498	568	70	756	835	80	670	670	0	1.878	1945
68		09AC001	Takhini River neat Whitehorse	60.9	-135.7	7050	1949	278	280	2	138	153	14	417	433	16	382	399	17	0.632	
69		10CD001	Muskwa River near Fort Nelson	58.8	-122.7	20300	1960	345	332	-13	177	189	11	522	521	-2	453	471	18	0.690	
70	60	11AA005	Milk River at Milk River	49.1	-112.1	2722	1912	104	108	5	245	242	-3	348	351	2	978	982	4	0.907	1947

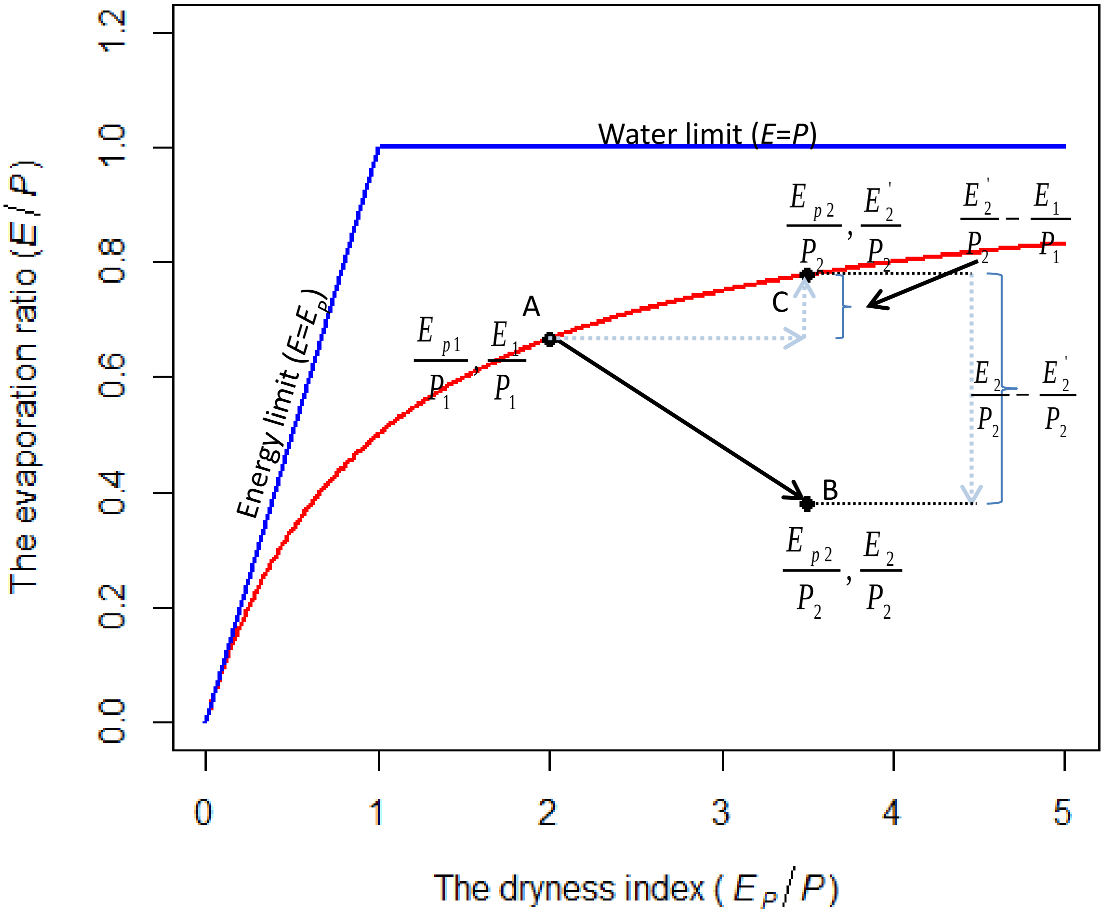

Supplementary Fig. S1. Typical Budyko curve (red line, *n*=1) and the schematic of decomposition method. Assuming point A is the catchment water balance under the stationary condition of the pre-change period (period-1), point B is under another stationary condition of the post-change period, and point C is a hypothetical point under a stationary condition which has the same catchment property as point A and the same climate condition (including precipitation and PET) as point B.

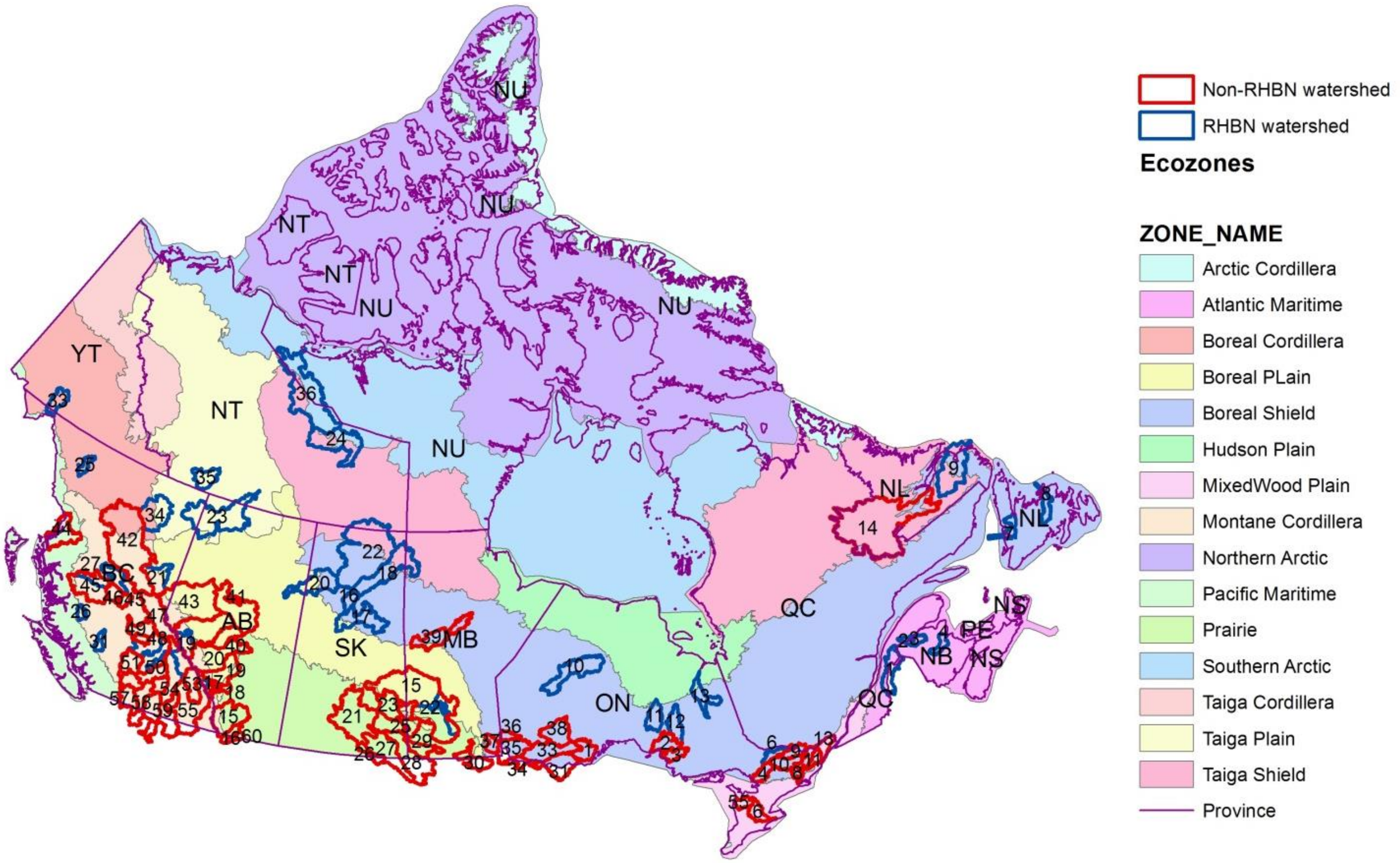

Supplementary Fig. S2. Geographic locations of the 96 studied drainage watersheds (polygons). The 15 terrestrial ecozones for Canada's landmass are also shown. The watershed number and the mean annual hydroclimatic variable values are shown in detail in the Tables S1 and S2. The map in Fig. S2 was generated with licensed ArcGIS 10.2 using public domain geographic data, such as the Atlas of Canada 1,000,000 National Frameworks Data (http://geogratis.gc.ca/) and ecozone data of the Ecological Framework of Canada (http://www.ecozones.ca/english/).

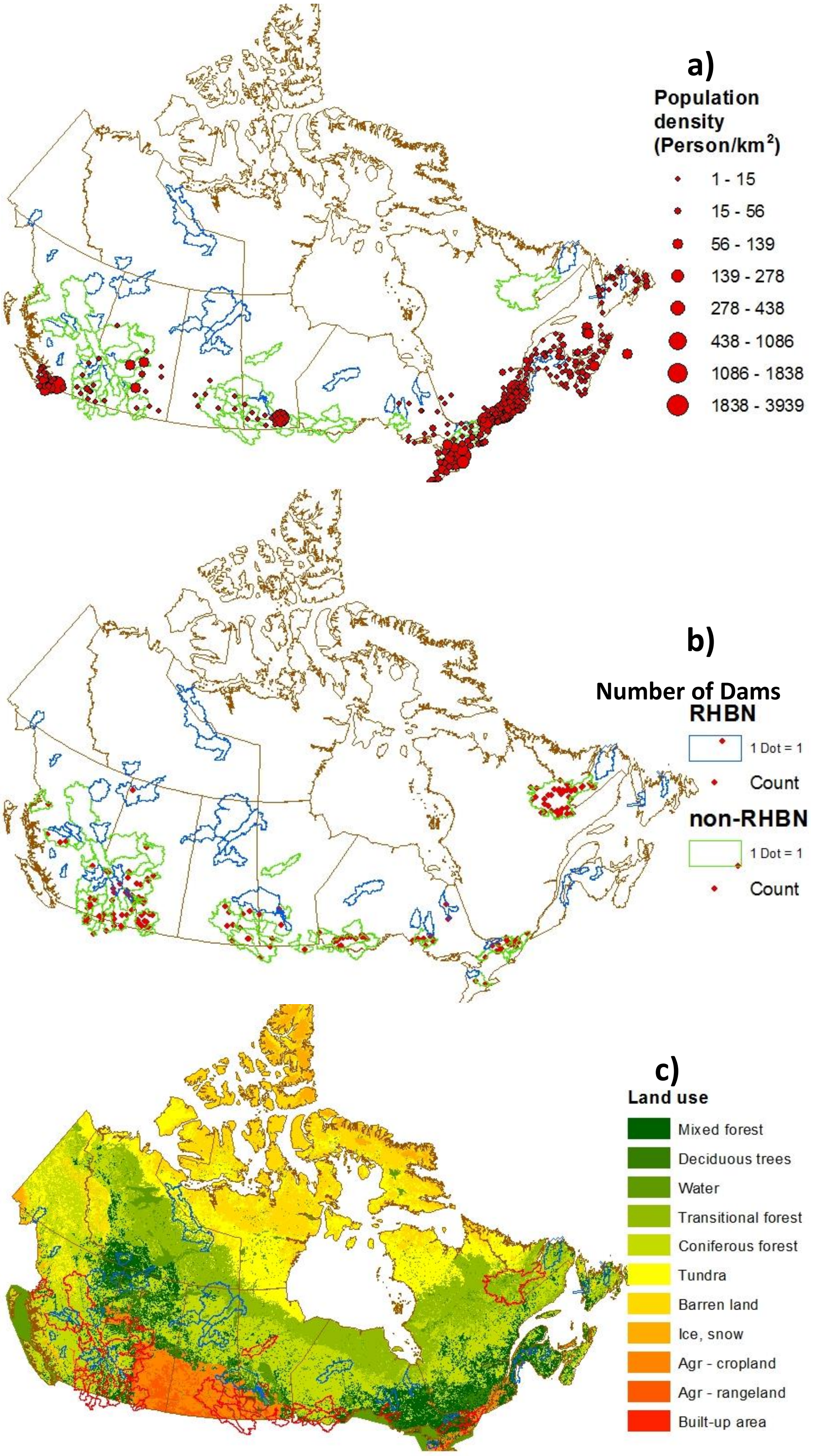

Supplementary Fig. S3. Spatial coverage of exogenous information used as a validation of the landscape change impacts due to human activities on mean annual streamflow, a) population density, b) number of dams in each watershed, and c) land use and cover. Maps in Fig. S3 were generated with licensed ArcGIS 10.2 using public domain geographic data (http://geogratis.gc.ca/).

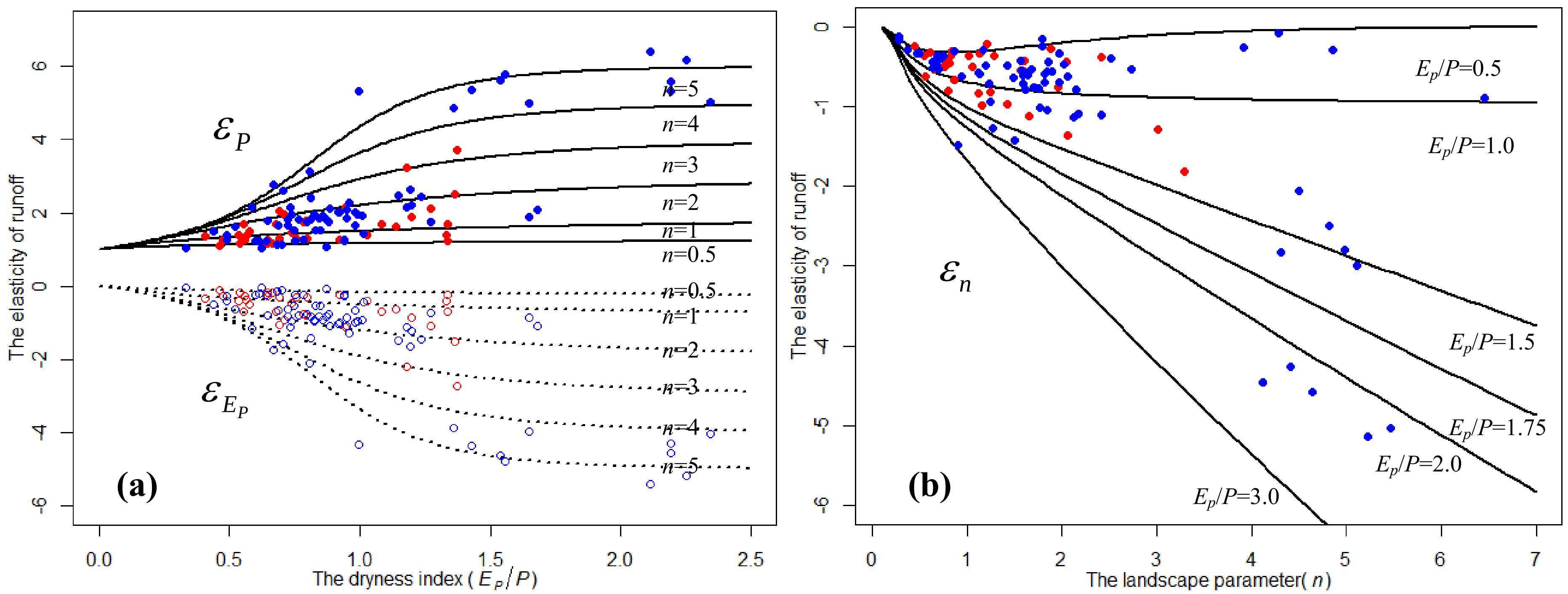

Supplementary Fig. S4. The elasticity of streamflow dependent on the dryness index (E_p/P) and the landscape parameter (n). The lines represent the elasticity of runoff, and the blue and red circles or dots represent the non-RHBN and RHBN watersheds, respectively.

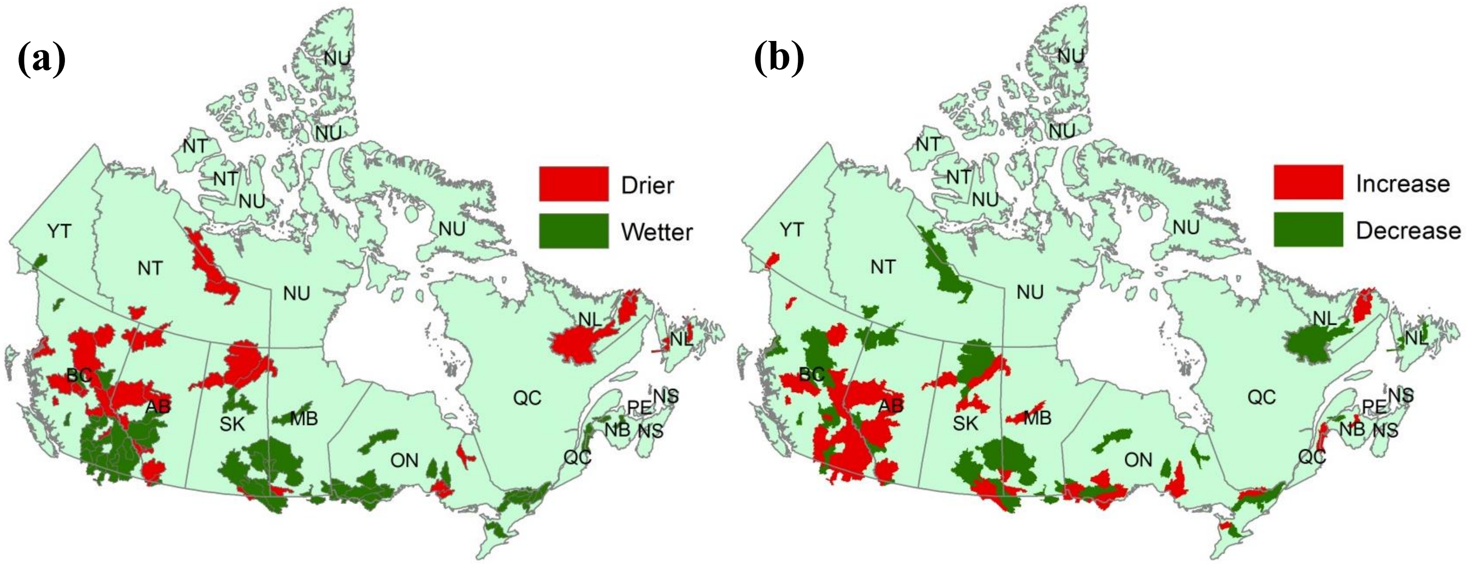

Supplementary Fig. S5. Difference in (a) the dryness index and (b) the evaporation ratio between the period-1 and period-2. Red (green) watersheds in (a) were getting drier (wetter) as the dryness index during period-2 was higher (lower) than that during period-1. Red (green) watersheds in (b) show an increase (decrease) in the evaporation ratio as the evaporation ratio during period-2 was higher (lower) than that during period-1. Maps in Fig. S5 were generated with licensed ArcGIS 10.2 using public domain geographic data (http://geogratis.gc.ca/).

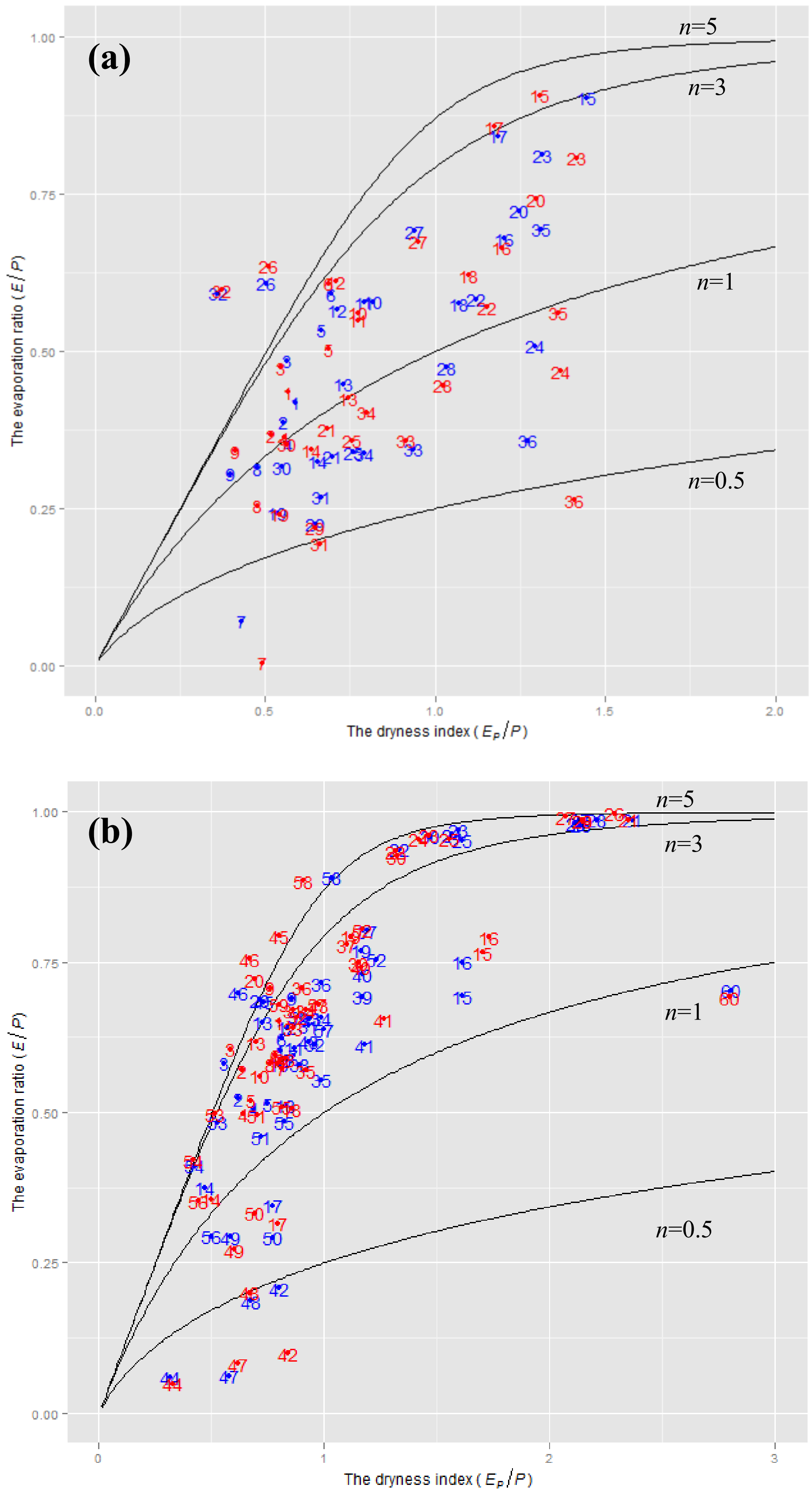

Supplementary Fig. S6. Distribution of the mean annual evaporation ratio (E/P) versus mean annual dryness index (E_0/P) for selected RHBN (a), and non-RHBN (b) watersheds during the period-1(blue dots) and period-2 (red dots). The Budyko curves calculated by Equation (2) are plotted as black solid lines.

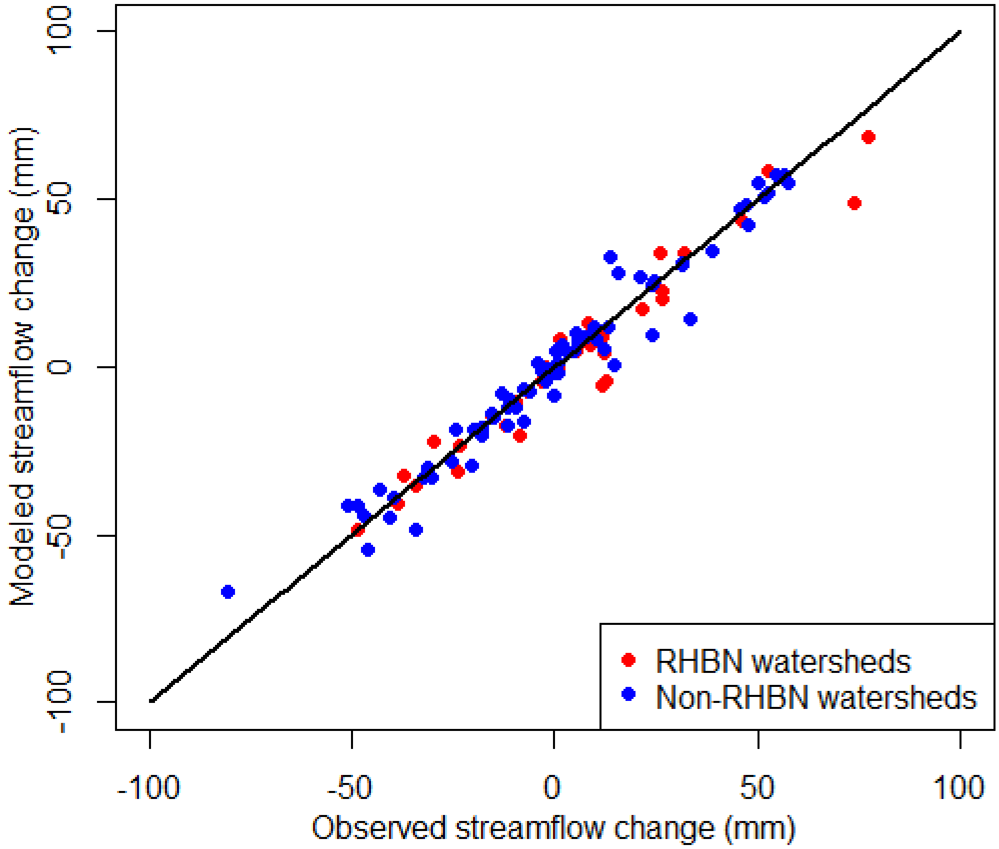

Supplementary Fig. S7 Comparison between the modeled and the observed streamflow change, the black solid line is a 1:1 straight line.

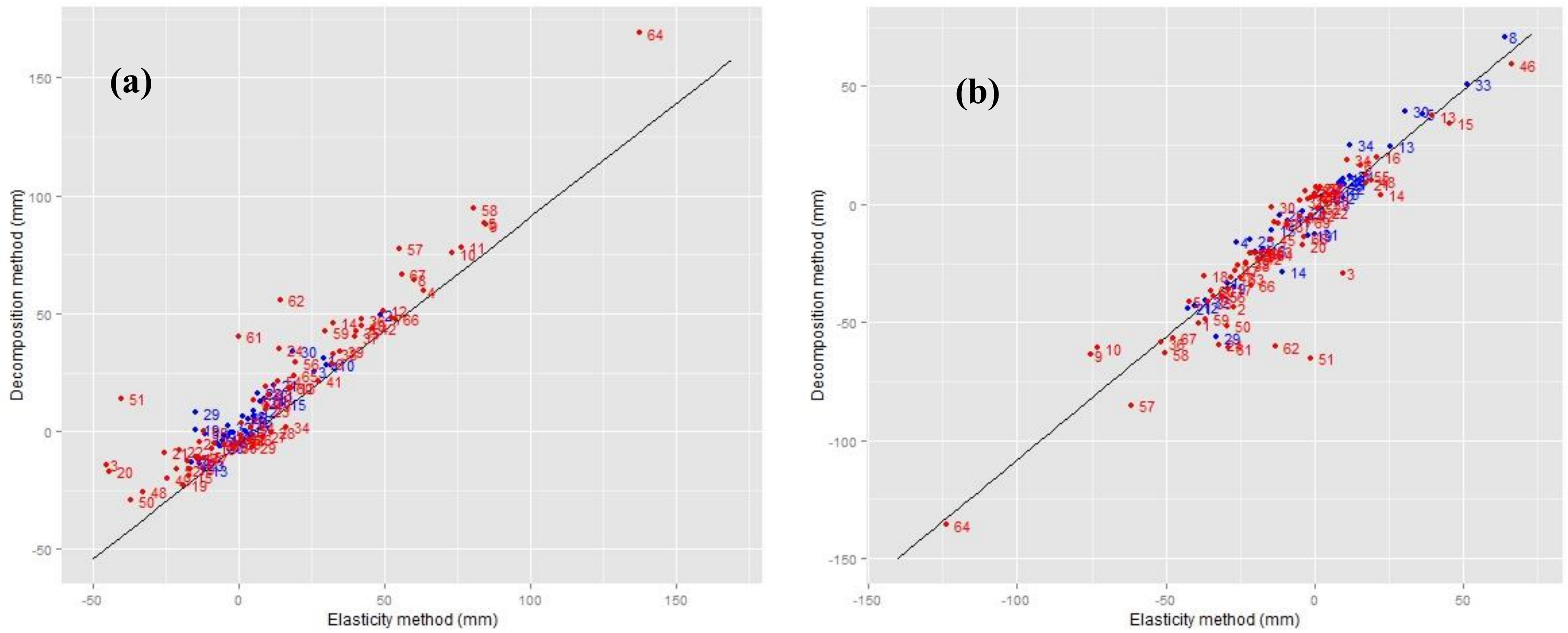

Supplementary Fig. S8. Comparisons of the contribution of climate (a) and human activities (b) to the streamflow change for watersheds described in Table S1 (blue dots) and S2 (red dots) derived from the decomposition method and the elasticity method.

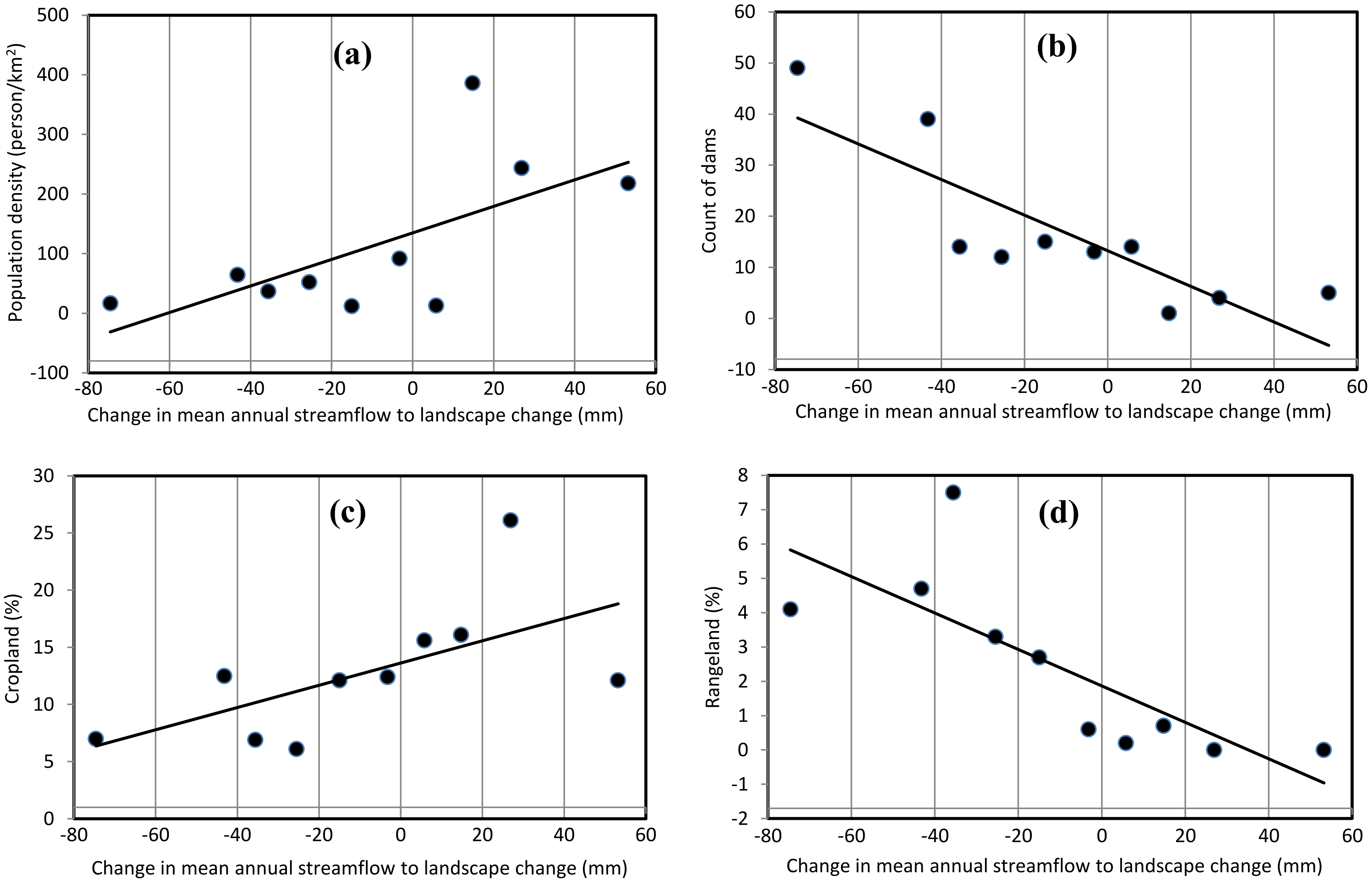

(a)
Population density (person/km²)
500
400
300
200
100
0
-100
-80
-60
-40
-20
0
20
40
60
Change in mean annual streamflow to landscape change (mm)
(b)
Count of dams
60
50
40
30
20
10
0
-10
-80
-60
-40
-20
0
20
40
60
Change in mean annual streamflow to landscape change (mm)
(c)
Cropland (%)
30
25
20
15
10
5
0
-80
-60
-40
-20
0
20
40
60
Change in mean annual streamflow to landscape change (mm)
(d)
Rangeland (%)
8
7
6
5
4
3
2
1
0
-1
-2
-80
-60
-40
-20
0
20
40
60
Change in mean annual streamflow to landscape change (mm)

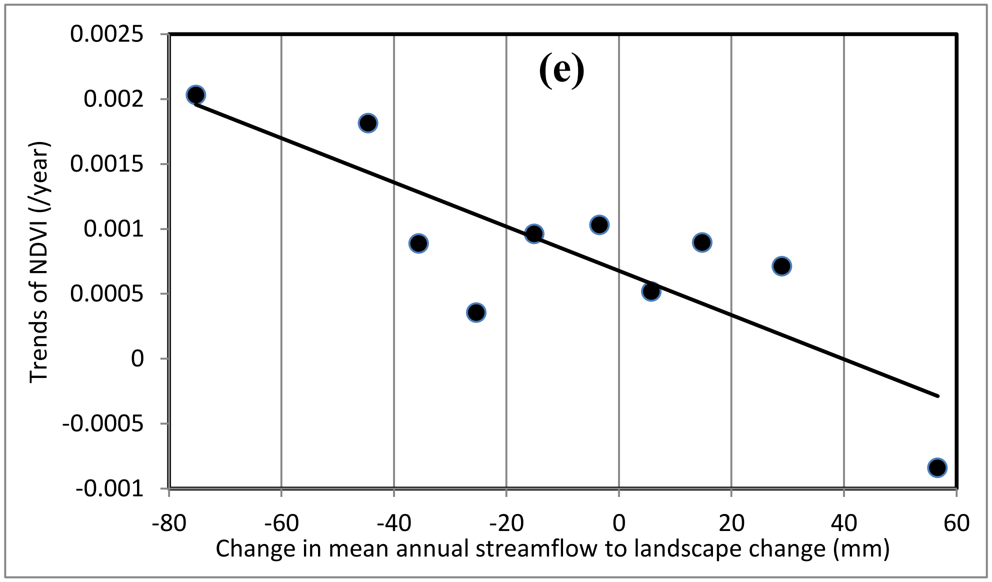

Supplementary Fig. S9. Relationships between estimated human contributions to streamflow change in terms of selected, external validation data, namely, (a) population density, (b) number of dams, (c) percentage of cropland, (d) percentage of irrigated land and (e) trend magnitudes of NDVI.

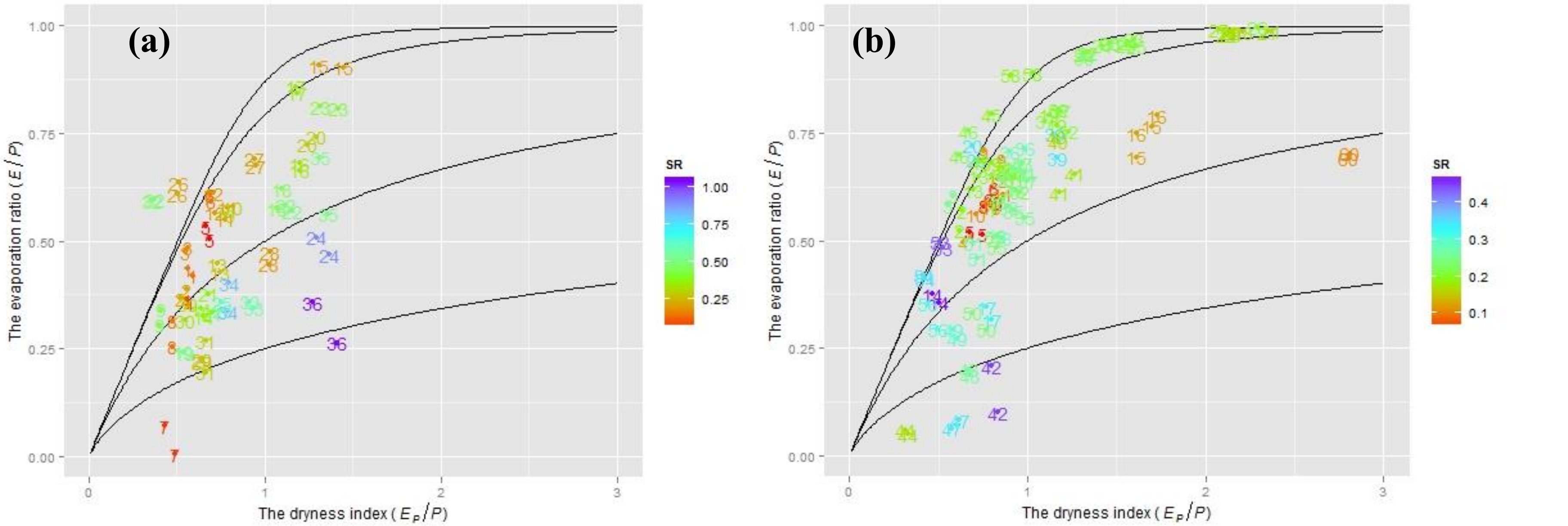

Supplementary Fig. S10. Same as Supplementary Fig. S6, but based on the snow ratio for selected RHBN (a) and non-RHBN (b) watersheds during period-1 and period-2 (see Fig. S6 about the period of data points). The Budyko curves calculated by Equation (2) are plotted as black solid lines.

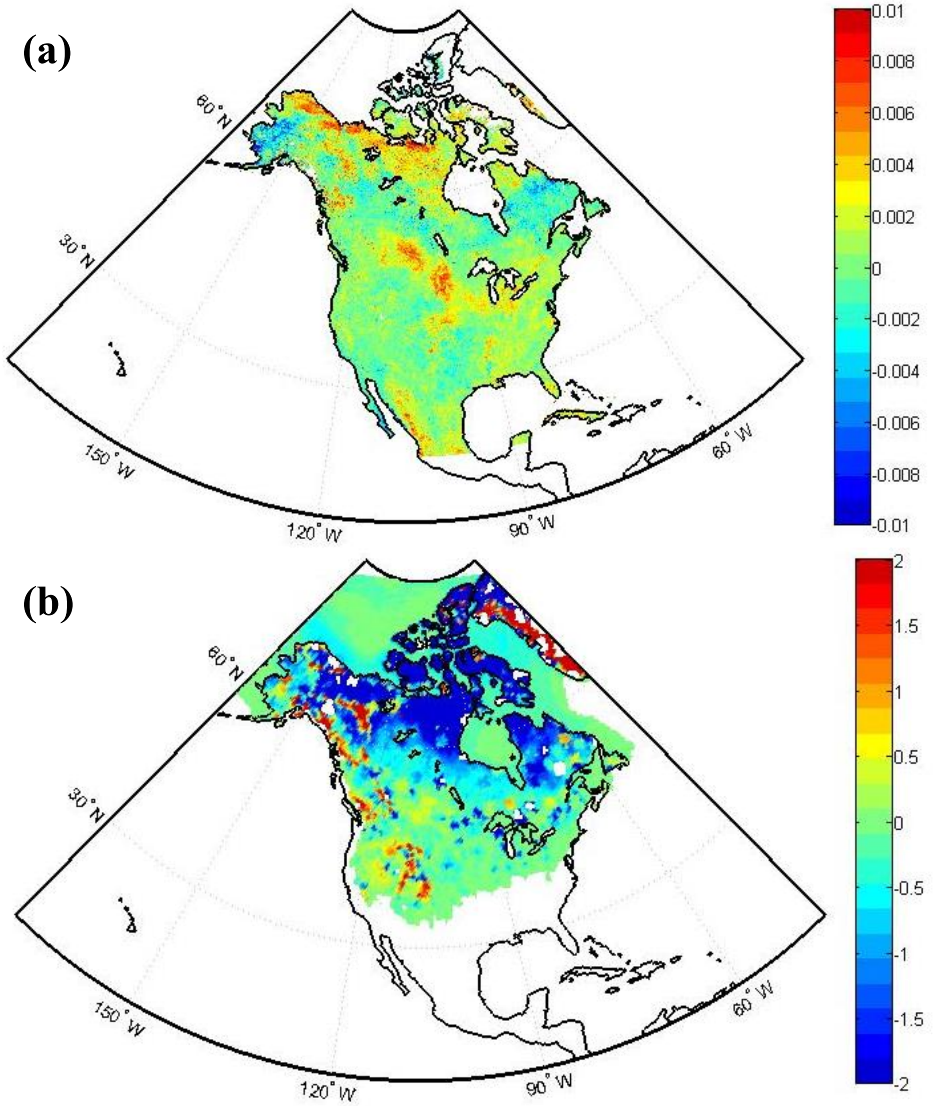

Supplementary Fig. S11 Trends of NDVI (a, year^{-1}) and snow ratio (b, % year^{-1}) over North America. Maps in Fig. S11 were generated with licensed Matlab R2014a using public domain data, such as NDVI data of the Global Inventory Modeling and Mapping Studies (GIMMS) (http://staff.glcf.umd.edu/sns/branch/htdocs.sns/data/gimms/) and snow ratio data of the North American Regional Reanalysis (NARR) (http://www.esrl.noaa.gov/psd/data/gridded/data.narr.html).
